# An experience of virtual leadership development for human resource managers

**DOI:** 10.1186/1478-4491-7-1

**Published:** 2009-01-08

**Authors:** Karen E Sherk, Fiona Nauseda, Sarah Johnson, Delphine Liston

**Affiliations:** 1 Management Sciences for Health, Cambridge, MA, USA

## Abstract

**Problem:**

Strong leadership and management skills are crucial to finding solutions to the human resource crisis in health. Health professionals and human resource (HR) managers worldwide who are in charge of addressing HR challenges in health systems often lack formal education in leadership and management.

**Approach:**

Management Sciences for Health (MSH) developed the Virtual Leadership Development Program (VLDP) with support from the United States Agency for International Development (USAID). The VLDP is a Web-based leadership development programme that combines face-to-face and distance-learning methodologies to strengthen the capacity of teams to identify and address health challenges and produce results.

**Relevant changes:**

The USAID-funded Leadership, Management and Sustainability (LMS) Program, implemented by MSH, and the USAID-funded Capacity Project, implemented by IntraHealth, adapted the VLDP for HR managers to help them identify and address HR challenges that ministries of health, other public-sector organizations and nongovernmental organizations are facing.

**Local settings:**

Three examples illustrate the results of the VLDP for teams of HR managers:

1. the Uganda Protestant and Catholic Medical Bureaus

2. the Christian Health Association of Malawi

3. the Developing Human Resources for Health Project in Uganda.

**Lessons learnt:**

The VLDP is an effective programme for developing the management and leadership capacity of HR managers in health.

## Introduction

The articles in this series on leadership and human resources (HR) have demonstrated the critical role that effective leadership plays in transforming HR strategies into results on the ground. It is essential that groups like the Global Health Workforce Alliance and the World Health Organization (WHO), as well as agencies such as the United States Agency for International Development (USAID) and other donors continue to provide leadership on a global level.

## Discussion

### Building HR leadership and management at all levels of the health system

How can we rapidly build HR leadership and management capacity at all levels of the health system? One approach to meet this challenge that has demonstrated promising results is the Virtual Leadership Development Program (VLDP). Developed by Management Sciences for Health (MSH) with support from USAID, the programme has been delivered to more than 1900 participants in more than 45 countries in Africa, Asia, the Eastern Mediterranean and Latin America and the Caribbean as of December 2008. Its premise is simple: bring teams of managers together and give them the skills and tools to scan their environments, focus on priority challenges, align and mobilize resources, address challenges and produce results.

Around the world, health professionals and HR managers are leading and managing public and private health organizations and systems with little or no formal management and leadership education and experience. Because strong management and leadership skills are crucial to finding solutions to the HR crisis, this is a gap that must be addressed.

Traditional ways to build capacity to lead change, such as sending an individual to an off-site workshop or course, can be slow and costly, and may disrupt services. Other disadvantages of traditional approaches include a theoretical rather than practical focus and participation of too few staff from the same organization in such training. Finally, when people are trained individually, they may find it difficult to generate support for change when they return to their workplaces.

### The Virtual Leadership Development Program

The VLDP is a Web-based programme that strengthens the capacity of teams to identify and address health challenges and produce results. It is available in Arabic, English, French, Portuguese, Russian and Spanish. Rather than giving a few senior managers off-site leadership training for one or two weeks, the VLDP trains teams via the Internet over the course of 13 to 16 weeks.

Adapted for HR managers and their teams and health services teams working on HR issues, the VLDP for HR managers focuses on identifying and addressing HR challenges. During the programme, each team strengthens its leadership capacity by applying MSH's leadership principles and practices and a model of challenge, feedback and support.

The VLDP requires approximately four hours of individual commitment per week. Team members work independently on the VLDP Web site with the support of a printed workbook. They also participate in on-site team meetings throughout the programme. During the VLDP, all the teams plan and develop a leadership project that addresses a real organizational challenge.

In 2006 and 2007, the Capacity Project, funded by USAID and implemented by IntraHealth, joined the USAID-funded Leadership, Management, Sustainability (LMS) Program implemented by MSH to deliver two VLDPs to teams of HR managers from different levels in ministries of health, private hospitals, universities, NGOs, and faith-based organizations in Ethiopia, Ghana, Kenya, Lesotho, Madagascar, Namibia, Nigeria, Rwanda, South Africa, Tanzania, and Uganda. The two programmes were jointly facilitated by a team of facilitators from MSH, IntraHealth and the Eastern and Southern Africa Management Institute (ESAMI). The challenges identified by the teams during the VLDPs spanned the gamut of HR issues, from staff retention to the need for good HR information.

### The impact of the VLDP on HR managers

Follow-up with the teams that participated in the VLDPs for HR managers took place in April and November 2007. Interviews with representatives from the teams revealed that teams are:

• beginning to demonstrate results as they implement their VLDP action plans to address the HR management challenges they identified;

• applying the concepts they learnt in the programme and using leadership and management tools in their organizations;

• working better in their organizational teams.

Examples of teams' progress as of November 2007 appear below.

### The Uganda Protestant and Catholic Medical Bureaus (UPMB and UCMB)

The UPMB and UCMB are umbrella organizations that oversee faith-based, private, not-for-profit health facilities in Uganda. During the first HR managers' VLDP, the team in Uganda identified actions to address their challenge of "retaining qualified health workers in the private not-for-profit health facilities." The activities included holding a consultation workshop with the health facility managers and designing and implementing a study to collect data about staff attrition. The study data will be used to develop retention strategies. The respondent reported that the team held the workshop, developed a survey and is collecting data. In July 2007, the team reported that they had implemented several staff-retention strategies. They have harmonized data collection instruments between the Protestant and Catholic Medical Bureaus in order to regularly monitor information such as personnel data, using these data to observe trends and take steps to reduce attrition. They have also coordinated with the Ministry of Health (MOH) on the hiring and placing of MOH staff with their organization, which has helped with retention and consistency. They have identified and learnt to better advertise the benefits of working for their organization. They say these changes were possible because of the leadership skills they gained through the VLDP.

### The Christian Health Association of Malawi (CHAM)

CHAM is a nonprofit, nongovernmental umbrella organization that oversees 167 member health units in all districts of Malawi. While participating in the second HR managers' VLDP, CHAM identified the following challenge: "How can we attract and retain qualified health personnel in three remote health facilities?"

The team outlined activities to address this challenge, including the installation of solar electricity in the facilities, service-level agreements for site renovations and development of staff incentive packages. In November 2007, a respondent from CHAM reported that the team has installed two Japan International Cooperation Agency (JICA)-donated solar energy systems in two of the three remote sites. Because of discussions held during the VLDP they were able to identify the need for the solar energy systems in remote sites as opposed to the planned installation closer to cities. Other progress includes the construction of two new homes near the remote sites; the establishment of three renovation service agreements; and collection of regional data by the team for the development of incentive packages.

### Developing Human Resources for Health (DHRH) in Uganda

DHRH is a European Union-supported, five-year project focusing on strengthening HR for health in Uganda. In November 2007, a respondent from the DHRH team in Uganda reported that the team has made important progress in addressing its challenge of improving the learning environment and opportunities for developing clinical laboratory skills among clinicians and medical and nursing students. The actions identified included establishing four skills laboratories in a national referral hospital and a school of nursing and identifying and training professional mentors. The respondent reported that skills laboratories have been established in 12 schools and medical institutions, and the team completed the training of 15 mentors and developed a training guide for training future mentors

## Conclusion

The VLDP is a model for strengthening the leadership capacity of all staff who have some responsibility for human resource issues in the health sector. It provides a way to upgrade the HR skills of the legions of personnel administrators and managers responsible for human resources and give them the confidence and capacity to implement solutions.

## Competing interests

The authors declare that they have no competing interests.

## Authors' contributions

SJ and KES oversee the implementation of all VLDPs for MSH. FN and DL managed the implementation of the two VLDPs discussed in this article. FN and DL also managed and completed the follow-up with VLDP teams discussed in this article. KES drafted the manuscript and SJ reviewed and edited it. All authors read and approved the final manuscript.

